# Single-Beam Sonar Motion Deformation Compensation and Localization Method for Underwater Robots in Confined Waters

**DOI:** 10.3390/s26175376

**Published:** 2026-08-25

**Authors:** Tianhong Ding, Zhiqiang Xu, Xiangyong Liu

**Affiliations:** 1 The Fishery Machinery and Instrument Research Institute, Chinese Academy of Fishery Science, Shanghai 200092, China; 2 East China Sea Fisheries Research Institute, Chinese Academy of Fishery Sciences, Shanghai 200090, China

**Keywords:** underwater robot, single-beam mechanical scanning sonar, spatiotemporal deformation compensation, underwater SLAM, multipath suppression, confidence weighting

## Abstract

In confined waters such as cylindrical aquaculture cages and ponds, underwater robots for cleaning, harvesting and other tasks that use single-beam mechanical scanning sonar for positioning and navigation are susceptible to multipath interference in complex water environments. Meanwhile, under extreme sea conditions, the severe attitude swaying of the robot and the slow-scanning characteristic of the sonar superimpose on each other, causing range stretching and helical deformation of the acoustic point cloud. To address these problems, this paper analyzes the deformation mechanism of single-beam sonar and proposes a spatiotemporal joint deformation compensation and localization-mapping method. First, an attitude-derived probabilistic confidence model is introduced as a lightweight robustness safeguard to characterize the geometric reliability of sonar echoes and reduce the contribution of low-confidence measurements during subsequent registration. Second, a beam-level spatiotemporal joint de-deformation algorithm is designed: the slant range in polar coordinates is flattened to eliminate nonlinear swaying deformation, and a beam-level displacement back-estimation based on the beam time offset and feedback velocity is employed to remove helical misalignment, thereby enhancing the underlying correction capability for dynamic deformation processes. Finally, a lightweight SLAM architecture that integrates keyframe-based dynamic sub-maps is constructed, where a confidence-weighted ICP is used to estimate the planar position with the heading provided by the compass and provide velocity-based closed-loop feedback, effectively mitigating the problem of global matching divergence caused by underlying dynamic deformations. Real-data-driven semi-physical disturbance tests based on measured pool data show that, under the injected ±45° roll disturbance and translational drift, the proposed method reduces the maximum point-to-reference error MaxAE from 1.059 m to 0.098 m. The reported mean internal registration residual decreases from 0.275 m for the traditional navigation odometry SLAM to 0.158 m for the proposed method, corresponding to a numerical reduction of approximately 42.5%. Under the evaluated conditions, the proposed method effectively mitigates point-cloud deformation and registration instability caused by robot swaying and slow-scanning sonar, while confidence weighting is retained as an auxiliary robustness mechanism for handling low-confidence correspondences.

## 1. Introduction

In modern aquaculture, underwater robots are increasingly used for net cage inspection, pool wall cleaning, and assisted harvesting, which is of great significance for improving the level of smart fishery equipment and reducing human risks [1,2]. The prerequisite for a robot to perform tasks such as cleaning and harvesting underwater is to accurately obtain its own pose and to construct the spatial structure of the surrounding environment. However, in actual aquaculture water bodies, due to fish activity, excrement, and bait feeding, the water quality is often highly turbid, making conventional optical vision sensors difficult to operate properly. Single-beam mechanical scanning imaging sonars such as the Echologger MRS900 are small, low-power, and highly penetrable, making them suitable for deployment on underwater robots and becoming a practical perception device in turbid underwater environments [3,4].

In recent years, extensive research has been conducted on acoustic positioning and perception for underwater robots both domestically and internationally, which can be divided into traditional acoustic beacon localization and sonar-based SLAM (simultaneous localization and mapping) methods. Traditional acoustic positioning methods such as long-baseline (LBL) and ultra-short-baseline (USBL) are relatively mature, but they rely heavily on pre-deployed beacon arrays. Such methods not only incur high deployment costs and long setup times, but are also prone to multipath errors in environments with severe acoustic reflections, such as aquaculture net cages or enclosed pools, resulting in poor adaptability [5,6,7,8]. To eliminate the dependence on external beacons, sonar-based SLAM technology has gradually become mainstream [9]. Researchers such as Burguera have used mechanical scanning sonars to build underwater maps and verified the effectiveness of scan matching in suppressing drift errors [10]. However, most of these studies have been validated in relatively static and open environments such as docks and bridge piers, while research targeting confined and dynamic scenarios like aquaculture is still lacking. In confined waters such as cylindrical net cages and harvesting ponds, underwater robots are susceptible to water current disturbances or cable tugs, leading to attitude changes and position drift. Since a mechanical scanning sonar typically takes several seconds to complete a full 360° scan, this “slow-scanning” characteristic, together with the continuous spatial motion of the robot, induces severe motion deformation in the sonar images. To address motion-estimation problems associated with asynchronously acquired sensor measurements, Furgale et al. formulated batch state estimation in continuous time using temporal basis functions, allowing a compact trajectory representation for high-rate and sweeping sensors [11]. Wu et al. developed a beam-level motion compensation framework for synthetic aperture sonar [12]. Compared with the continuous-time temporal-basis-function formulation of Furgale et al. [11], the method proposed in this study employs a lightweight velocity-prior feedback loop for beam-level displacement compensation, avoiding batch estimation of spline coefficients and facilitating deployment on low-power underwater robots. Furthermore, unlike Wu et al. [12], which focuses on signal-level phase compensation for multi-hydrophone arrays, our approach specifically targets the macroscopic geometric deformation (e.g., helical misalignment) unique to the extremely slow-scanning mechanism (approximately 7.9 s/scan) of single-beam mechanical sonars. Nevertheless, when facing the complex operating environment of real aquaculture net cages [13,14], existing methods still suffer from problems such as a single compensation dimension and matching divergence caused by multipath false targets when dealing with the complex sonar deformation jointly induced by “nonlinear swaying” and “temporal translation” in confined waters.

To address the above problems, this paper proposes a novel, tightly coupled spatiotemporal joint deformation compensation and localization method for single-beam sonar in confined waters. Most importantly, rather than a simple sequential combination of existing algorithms, the proposed architecture physically couples 3D attitude with acoustic multipath characteristics to form a closed-loop system. The method deeply analyzes the projection deformation mechanism of sonar polar coordinates in three-dimensional space. First, an attitude-derived probabilistic weighting model is introduced to characterize the geometric confidence of each retained sonar beam. Rather than being treated as the primary source of mean-error reduction, the resulting weight is embedded into the Weighted ICP back-end as a lightweight robustness safeguard that reduces the contribution of low-confidence correspondences when such measurements occur. Second, a beam-level spatiotemporal joint deformation compensation method is proposed to simultaneously eliminate the nonlinear stretching of ranging and the helical misalignment of point clouds. Finally, a lightweight weighted SLAM architecture integrating keyframe-based dynamic sub-maps is constructed, where the aforementioned confidence weight is directly embedded into the Weighted ICP backend, and the output velocity is fed back to the temporal compensation module. From low-level physical correction to high-level algorithmic loop closure, this method provides more reliable localization and mapping for underwater robots in turbid, vision-deficient environments.

## 2. Underwater Robot Platform and Sonar Deformation Mechanism

### 2.1. Robot Experimental Platform and Sensor Configuration

**Figure 1:**
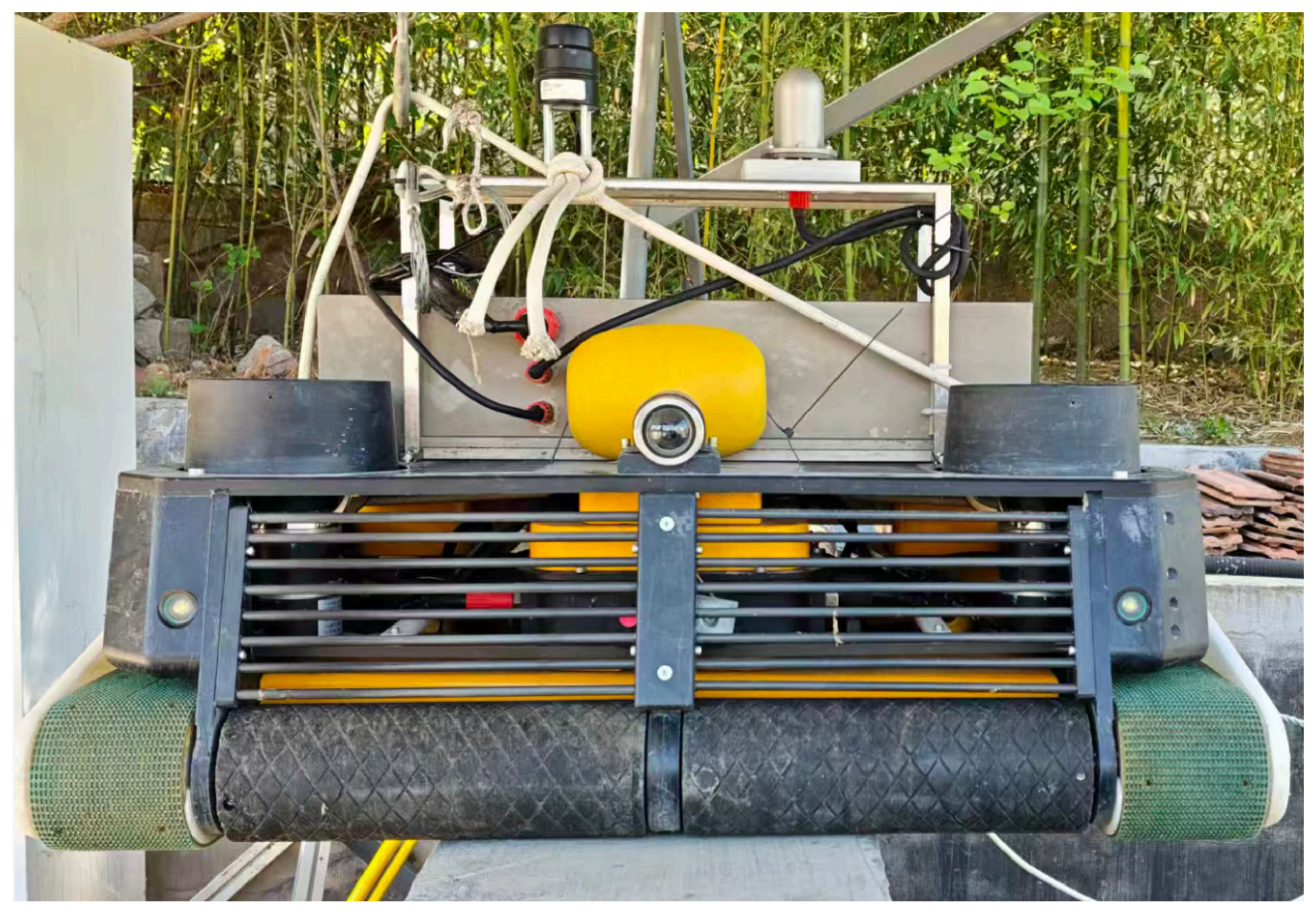
Underwater robot experimental platform.

The experimental platform used in this paper is a self-developed frame-type underwater robot, with its overall structure shown in Figure 1. The robot is designed with good power capability and can carry various types of perception equipment. In terms of environmental perception and self-pose measurement, it is mainly equipped with two types of core sensors. The first is the Echologger MRS900 single-beam mechanical scanning imaging sonar (EofE Ultrasonics Co., Ltd., Goyang-si, Gyungki-do, Korea). Its working principle is to drive the acoustic transducer to rotate 360° in the horizontal plane through an internal stepper motor and to obtain the environmental distance using the time-of-flight principle. In the profiling mode, the step angle is 1.6° per step, and it usually takes several seconds to complete a full scanning cycle. This ”slow-scanning” characteristic is the main source of temporal motion deformation. The second is a compass attitude measurement device(Beijing Wisensing Technology Co., Ltd., Beijing, China), which outputs the robot’s yaw, pitch, and roll angles. Because the sonar ranging frequency and the compass attitude output frequency differ significantly, this paper develops a timestamp-based soft synchronization mechanism. For each sonar beam timestamp, the temporally nearest compass measurement within a tolerance of 50 ms is associated with the beam, and unmatched attitude values are filled using adjacent valid measurements, thereby obtaining the corresponding three-dimensional attitude for subsequent beam-level motion compensation.

### 2.2. Coordinate System Definition and Motion Deformation Mechanism

To quantify the deformation patterns, this paper defines two fundamental spatial coordinate systems. The first is the sonar local polar coordinate system *S*(*d*,*a*), which takes the sonar transducer center as the origin, where *d* is the acoustic echo distance and *a* is the mechanical scanning angle. The second is the robot body rectangular coordinate system *B*(*x*,*y*,*z*), where the forward direction of the robot is defined as the *y*-axis, the rightward direction as the *x*-axis, and the vertically downward direction as the *z*-axis. Under ideal conditions, if the robot remains perfectly horizontal and stationary, the sonar data in the polar coordinate system can be directly mapped to the two-dimensional plane through standard trigonometric functions. However, in real dynamic operating environments, the sonar point cloud undergoes deformation.

The first issue is the nonlinear range stretching caused by attitude swaying. When the robot pitches or rolls due to water current disturbances or cable tugs, the beam originally designed to be parallel to the horizontal plane is inclined and strikes the pool wall or net cage at a certain angle. In this case, the measured sonar distance is actually the hypotenuse in three-dimensional space, which is larger than the true horizontal distance. If this inclined projection is ignored and the two-dimensional mapping is directly applied, an originally straight pool wall will appear as an outwardly bulging curved deformation in the point cloud map.

In addition, there is the temporal misalignment problem caused by the superposition of slow scanning and translational motion. Assuming the robot moves forward at a speed V, by the time the sonar scans the i-th beam, a time interval Δti has elapsed, and the robot has actually moved forward a distance V*Δti. If all beams of one full scan are placed at the origin of the starting moment, the point cloud will be severely distorted, and a rectangular pool will be stretched into a spiral or a parallelogram shape.

### 2.3. Comparison Methods

For comparative purposes, this study selects four baseline methods: the traditional 2D static projection algorithm, the beam-level constant-velocity translational compensation algorithm, the 3D attitude compensation algorithm without temporal correction, and an adapted continuous-time cubic B-spline method based on Furgale et al. [11]. The details of each algorithm are as follows.

The traditional 2D static projection algorithm assumes that the platform’s position and attitude remain unchanged during the few seconds required for one complete sonar scan. It directly maps the echo distances measured in the sonar polar coordinate system to the two-dimensional plane using the pose at the start of the scan, without correcting the spatial range stretching caused by platform swaying or compensating for the temporal platform translation during the scan. This most primitive projection method is typically used when the underwater robot is not equipped with a compass or inertial measurement unit, or when no motion compensation is introduced. When Burguera et al. conducted high-resolution mapping using a mechanical scanning sonar, they used the uncompensated raw projection as a baseline and found that, without real-time pose information, the point cloud exhibited significant stretching and misalignment due to platform motion [3].

The beam-level constant-velocity translational compensation algorithm uses the estimated planar velocity and the time offset of each sonar beam to compensate for the translational displacement accumulated during one scan. This method corrects the temporal misalignment caused by platform translation but does not use pitch and roll measurements to correct attitude-induced slant-range stretching. In the subsequent single-scan compensation experiment, this baseline is referred to as beam-level constant-velocity translational compensation only. In the continuous trajectory experiment, the corresponding framework is referred to as traditional navigation odometry SLAM.

The 3D attitude compensation algorithm without temporal correction uses the pitch and roll measurements associated with each sonar beam to project the measured slant range onto the horizontal plane, thereby correcting the range stretching caused by platform swaying. However, this method does not compensate for the translational displacement accumulated during one complete scan, and temporal misalignment therefore remains in the resulting point cloud. In the subsequent experimental comparisons, this baseline method is referred to as 3D attitude compensation SLAM without temporal correction. The continuous-time cubic B-spline baseline is adapted from the temporal-basis-function formulation proposed by Furgale et al. [11]. The planar displacement during one complete sonar scan is represented by cubic temporal basis functions fitted to seven uniformly distributed pose observations. The estimated continuous displacement is evaluated at the acquisition time of each sonar beam and is used to correct the intra-scan translational misalignment after the same three-dimensional attitude flattening. Because the original method was not developed specifically for the present single-beam sonar data format, this baseline is referred to as an adapted implementation rather than an exact reproduction of the original system.

### 2.4. Experimental Scene and Basic Data Acquisition

**Figure 2:**
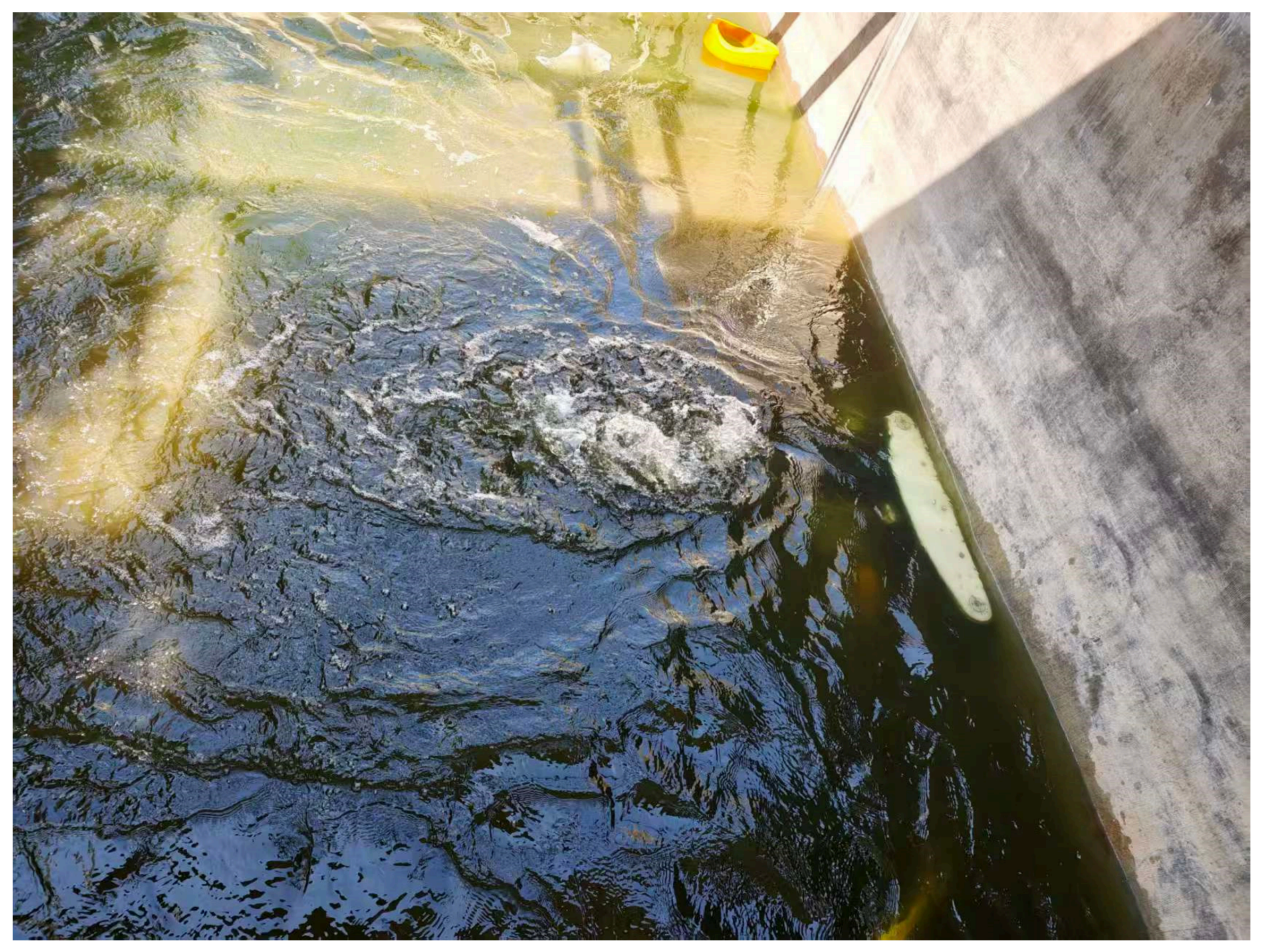
Underwater robot pool data acquisition.

To realistically reproduce the working environment of underwater robots and provide a reliable physical data foundation for subsequent algorithm verification, this paper adopts a data acquisition scheme based on a real pool environment. The experiments are conducted in a standard rectangular pool measuring approximately 6 m × 3 m, as shown in Figure 2, where the underwater robot platform equipped with a single-beam sonar is deployed. During data acquisition, the robot is controlled to perform complete sonar scans under relatively calm, still-water conditions to collect raw baseline datasets. These measured data truly record the hardware noise of the single-beam sonar in confined waters, the echoes from underwater suspended particles, and the severe acoustic multipath reflections caused by the pool walls. This measured dataset is used to evaluate the effectiveness of the front-end acoustic filtering and geometry-based validity screening and provides a measured-data basis for the real-data-driven semi-physical disturbance-injection tests presented in the subsequent sections.

## 3. Robot Localization Algorithm

### 3.1. Flowchart of the Localization Algorithm

**Figure 3:**
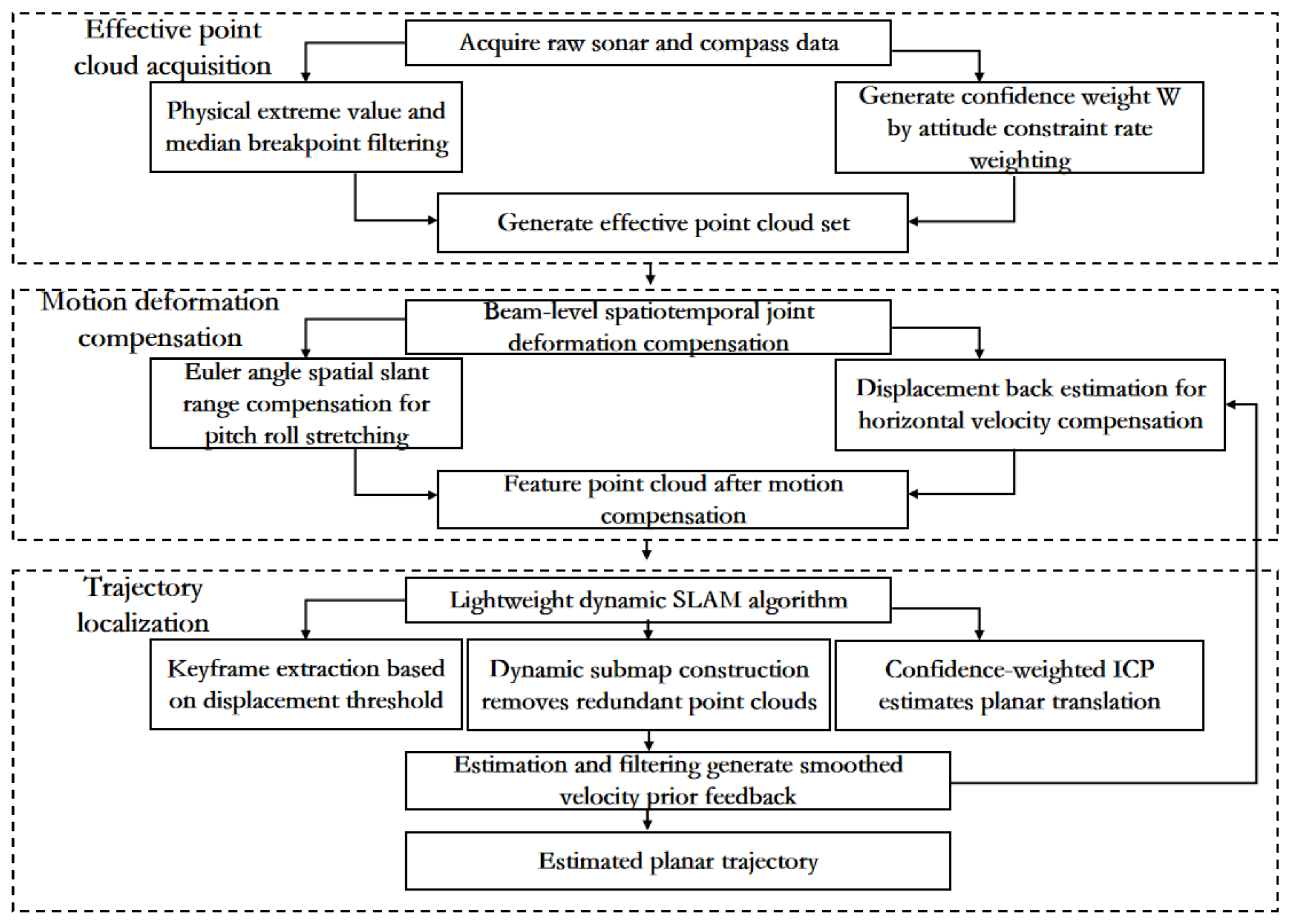
Overall flowchart of the localization algorithm.

To address the problems of single-beam mechanical scanning sonar for underwater robots in confined waters such as cylindrical aquaculture cages and harvesting ponds, where multipath interference and platform attitude disturbances are severe, this paper proposes a localization method. The overall technical roadmap is shown in Figure 3, which mainly consists of three modules: effective point cloud acquisition, motion compensation, and trajectory localization.

First, in the effective point cloud acquisition module, sonar ranging data and compass attitude data are obtained. The sonar operates in a profiling mode where its embedded firmware automatically detects the target reflection and directly outputs the calculated range value. Consequently, the raw point cloud is acquired directly from the predefined hardware output without requiring secondary peak detection algorithms. Time-stamp-based beam-level synchronization solves the asynchronous sampling problem between the sonar and the 20 Hz compass attitude data. To address the severe acoustic multipath interference in confined waters, physical extreme value and median filtering are first applied directly to this discrete sequence of calculated ranges rather than raw acoustic images to remove noise from suspended particles. Geometry-based validity screening is first applied to remove evidently unreliable measurements. A geometric confidence weight W is subsequently generated for each retained beam according to the deviation of its emission direction from the horizontal plane and is passed to the subsequent weighted registration. The weight serves as an auxiliary robustness term for reducing the contribution of geometrically low-confidence correspondences.

Next, the feature sequence is fed into the motion compensation module for beam-level spatiotemporal joint deformation compensation. The first step is spatial compensation: the data are mapped to the local polar coordinate system, and then the instantaneous roll and pitch angles at the beam emission time are used to perform spatial slant-range projection, accurately flattening the inclined range measurement to the horizontal plane. This effectively corrects the nonlinear spatial range stretching caused by platform swaying. The second step is temporal compensation: using the smooth velocity fed back from the back-end, the beam-level displacement is back-estimated according to the time offset of each beam, eliminating the helical temporal misalignment caused by the superposition of slow scanning and platform motion. The compensated feature point cloud is then obtained.

Finally, in trajectory localization, a lightweight dynamic SLAM algorithm is adopted. In the front-end, keyframes are extracted based on displacement thresholds, and dynamic local submaps are constructed, effectively removing redundant historical data generated when the robot hovers in place during pool wall operations, thereby improving computational efficiency and inference speed. In the back-end point-cloud registration stage, the confidence generated by the front-end is incorporated into a Weighted ICP solver to reduce the contribution of low-confidence correspondences during planar position estimation. After the planar position estimate is obtained, the observed planar velocity is calculated from the estimated inter-scan displacement and smoothed by exponential filtering. The smoothed velocity is then fed back as a prior state quantity to the front-end compensation module, forming a closed-loop correction network. Finally, the estimated planar trajectory of the robot is obtained.

### 3.2. Multipath Suppression and Probabilistic Weighting Method Based on 3D Attitude Constraints

When operating in confined waters such as ponds and aquaculture cages, the closed boundaries around them tend to cause specular reflection and multipath effects of acoustic waves. When the sonar range measurement is disturbed by multipath interference, a false ghost wall appears outside the real boundary. Traditional denoising algorithms usually set static pitch and roll thresholds for hard rejection. However, when the robot sways violently, hard thresholds may over-cut the effective feature points of the real pool wall, leading to insufficient matching constraints for subsequent SLAM. Therefore, this paper proposes a soft-confidence probabilistic weighting model based on three-dimensional spatial attitude constraints. According to the geometric characteristics of acoustic wave propagation, the closer the beam emission direction is to the absolute horizontal plane, the higher the probability of hitting the true side wall; conversely, the larger the deviation of a beam (aimed at the water surface or pool bottom) from the horizontal plane, the higher the risk of multipath reflection.

Let the mechanical scanning angle of the i-th beam be $\alpha_{i}$, and let $\theta_{i}$ and $\phi_{i}$ denote the pitch and roll angles associated with the beam timestamp through nearest-neighbor timestamp matching, respectively. From a geometric perspective, this elevation deviation is essentially the projection of the acoustic beam unit vector onto the absolute vertical axis. By applying a spatial rotation matrix to the beam directional vector and utilizing small angle approximations for the vehicle attitude, the vertical component is decoupled into a linear combination of the current pitch and roll. The spatial angle $\gamma_{i}$ by which this acoustic ray deviates from the absolute horizontal plane in three-dimensional space is derived through analytic geometry as:

(1) $$\gamma_{i}=\theta_{i}cos(\alpha_{i})+\phi_{i}sin(\alpha_{i})$$

To convert the multipath risk into a weight that can be used in subsequent optimization equations, a Gaussian decay function is introduced to generate a geometric confidence weight $w_{i}$ for each beam:

(2) $$w_{i}=max\left(exp\left(-\frac{\gamma_{i}^{2}}{2\sigma^{2}}\right),w_{min}\right)$$

where σ is a standard deviation constant controlling the decay width of the weight, and $w_{min}$ is a set anti-singularity minimum value to ensure that under extreme attitudes the feature points are not completely zeroed out, thereby preserving the geometric topological continuity. The selection of the Gaussian function over linear or standard exponential decay is strictly driven by the physical tolerance requirements for underwater robot swaying. The Gaussian curve inherently maintains a relatively flat peak near zero deviation. This characteristic perfectly preserves near-maximum confidence weights during typical small-angle operational sway. In contrast, linear or simple exponential models would inappropriately degrade the weights of effective boundary beams during normal stable navigation. Furthermore, the Gaussian function rapidly penalizes large tilt angles where the geometric risk of hitting the water surface or bottom increases nonlinearly. This probabilistic model assigns smaller optimization weights to beams with larger elevation deviations and is intended to reduce the influence of geometrically low-confidence measurements during subsequent registration. It should be noted that this probabilistic weighting model is primarily optimized for rigid vertical boundaries such as concrete ponds or breeding tanks. In actual flexible aquaculture cages, the multipath behavior is more complex and couples with the acoustic impedance of the net material, varying incidence angles, and dynamic distances to the water surface and bottom. In such unstructured environments, this elevation-based confidence weight serves as a conservative baseline filter, and the residual complex multipath artifacts are further suppressed by the subsequent robust matching back-end.

### 3.3. Beam-Level Spatiotemporal Joint Deformation Compensation Algorithm for Mechanically Slow Scanning

To eliminate the spatial swaying deformation and temporal translational misalignment of the robot from the physical root, this paper does not adopt the intra-frame static two-dimensional assumption. Instead, a beam-level spatiotemporal joint deformation compensation algorithm accurate to a single acoustic beam is proposed.

First, three-dimensional attitude spatial compensation is performed. For the polar coordinate system of the hardware, the 0° direction of the sonar is set to point to the robot’s forward direction (the positive *y*-axis) with clockwise rotation. The polar coordinates ($d_{\;i}$, $\alpha_{i}$) of the i-th beam are expanded into the forward component $y_{1,i}$ and the rightward component $x_{1,i}$ in the local coordinate system:

(3) $$y_{1,i}=d_{i}cos(\alpha_{i})\;\;\;x_{1,i}=d_{i}sin(\alpha_{i})$$

Next, the instantaneous roll angle $\varphi_{i}$ and pitch angle $\theta_{i}$ at the beam emission time are introduced. A three-dimensional space rotation matrix is used to perform nonlinear correction, projecting the acoustic slant range measured under tilt to the absolute horizontal plane. The projection process simultaneously handles the tilt caused by the superposition of pitch and roll:

(4) $$y_{2,i}=y_{1,i}cos\left(\theta_{i}\right)+x_{1,i}sin\left(\varphi_{i}\right)sin\left(\theta_{i}\right)x_{2,i}=x_{1,i}cos(\phi_{i})$$

The derivation of this spatial flattening assumes that the acoustic reflection boundary is strictly vertical. While this geometric projection perfectly recovers the structural contour in standard rectangular or cylindrical aquaculture pools, an inherent projection residual will occur when facing non-vertical or flexible inclined cage nets. Under such unstructured boundary conditions, the proposed algorithm relies on the dynamic sub-map and the confidence-weighted ICP to absorb these local projection residuals as geometric noise, ensuring global topological consistency rather than absolute local geometric fidelity. At this stage, the planar coordinates ($x_{2,i}$,$y_{2,i}$) eliminate the outward-bulging range-stretching deformation caused by attitude swaying. Then, temporal compensation is carried out. The yaw angle $\psi_{i}$ is incorporated to transform the coordinates to the global attitude reference:

(5) $$y_{3,i}=-x_{2,i}sin\left(\psi_{i}\right)+y_{2,i}cos\left(\psi_{i}\right)x_{3,i}=x_{2,i}cos\left(\psi_{i}\right)+y_{2,i}sin\left(\psi_{i}\right)$$

Finally, to eliminate the displacement deviation caused by slow scanning, the time delay $\Delta t_{i}$ between the emission time $t_{i}$ of this beam and the start time $t_{0}$ of the current scan is extracted. The smooth prior velocities $V_{x}$ and $V_{y}$ fed back from the back-end SLAM module are used to compensate the robot’s own translation:

(6) $$X_{i}=x_{3,i}+V_{x}\cdot \Delta t_{i}\;\;\;Y_{i}=y_{3,i}+V_{y}\cdot \Delta t_{i}$$

At the system startup phase, the initial prior velocities are strictly set to zero to mathematically mirror the stationary deployment state of the robot. Consequently, the temporal compensation term naturally zeros out for the initial frame, meaning the first point cloud undergoes purely three-dimensional spatial attitude flattening without translational correction.

### 3.4. Lightweight SLAM Algorithm with Dynamic Sub-Maps and Confidence-Weighted ICP

After obtaining the underlying deformation-free feature point cloud, a back-end matching architecture is constructed to eliminate long-term cumulative drift. Since the onboard computing power of underwater robots is limited and cleaning operations often involve redundant actions such as in-place hovering, traditional SLAM algorithms based on a single global map are prone to computational explosion and local extremum deadlock. Therefore, this paper designs a lightweight dynamic SLAM algorithm.

First, keyframe extraction and dynamic sub-map construction are performed based on a spatial threshold. Instead of frame-by-frame registration, a spatial displacement threshold ΔS is set. Only when the robot’s displacement increment exceeds ΔS is the current compensated point cloud stored as a new keyframe. When executing the next frame localization, the algorithm searches for historical keyframes within a fixed radius R around the prior position predicted by the constant-velocity model, and assembles them into a local sub-map. This dynamic cropping mechanism ensures that the number of point clouds involved in each matching operation remains constant, guaranteeing real-time responsiveness.

Next, a confidence-weighted ICP (Iterative Closest Point) solution with loop closure feedback is introduced. In the point cloud registration stage, the multipath confidence weight $w_{i}$ generated in Section 3.2 is incorporated into the ICP optimization objective function. Let $p_{i}^{src}$ be a feature point in the current frame and $p_{i}^{tgt}$ be its nearest neighbor in the local sub-map. Because the heading is provided by the compass, the registration process optimizes only the planar translation. The weighted translation is obtained by minimizing the following objective function:

(7) $$E(t)=\sum_{i=1}^{M}w_{i}{\left\|p_{i}^{src}+t-p_{i}^{tgt}\right\|}_{2}^{2}$$

The weighted centroids of the current scan and the corresponding local sub-map points are calculated as follows:

(8) $${\bar{P}}_{src}^{w}=\frac{\sum_{i=1}^{M}w_{i}P_{i}^{src}}{\sum_{i=1}^{M}w_{i}}\;\;\;{\overset{¯}{P}}_{tgt}^{w}=\frac{\sum_{i=1}^{M}w_{i}P_{i}^{tgt}}{\sum_{i=1}^{M}w_{i}}$$

After introducing the weights $w_{i}$, high-confidence correspondences contribute more to the estimated translation, whereas low-confidence correspondences contribute less. This weighting is intended to reduce the sensitivity of the registration solution to sporadic unreliable echoes. When most retained measurements already have weights close to one, the weighted solution naturally approaches standard ICP.

Once the current planar position estimate is obtained, the estimated inter-scan displacement is divided by the corresponding time interval to derive the observed planar velocities. To prevent occasional pose matching errors from causing cascading errors in the front-end motion compensation, an exponential moving average (EMA) filter is used for state update:

(9) $$V_{x}^{\left(k\right)}=\beta V_{x}^{\left(k-1\right)}+\left(1-\beta \right)v_{x}^{obs}\;\;\;V_{y}^{\left(k\right)}=\beta V_{y}^{\left(k-1\right)}+(1-\beta )v_{y}^{obs}$$

where β is the smoothing inertia coefficient. To resolve the apparent circular dependency between the front-end compensation and the back-end SLAM during initialization, a step-delay recursive feedback mechanism is implemented. Because the initial velocities are zero, the first keyframe is aligned and added to the root sub-map relying solely on the aforementioned spatial attitude correction. Once the robot initiates movement, the Weighted ICP solves for the displacement to generate the first observed velocity. The EMA filter then utilizes the smoothed velocity from the previous step to compensate for the incoming acoustic beams of the current step. The updated stable velocities *Vx*, *Vy* are fed back as prior state quantities to the displacement back-estimation formula in Section 3.3, thus forming a complete localization closed loop in which the back-end provides smoothed velocities and the front-end eliminates deformation accordingly.

## 4. Experimental Results and Analysis

### 4.1. Experimental Platform and Test Environment

In this study, an underwater robot test platform was set up in a rectangular pool measuring 6 m × 3 m with a water depth of 1.5 m. The robot used in the experiments is a frame-type underwater robot, which was controlled to operate at a mid-water depth of 0.8 m to minimize direct multipath reflections from the water surface and pool bottom. It is equipped with two types of core sensors. The first is an Echologger MRS900 single-beam mechanical scanning imaging sonar for environmental perception, mounted on top of the robot. The sonar operates at a center frequency of 850 kHz with a beamwidth of 2.0 degrees. To optimally cover the experimental pool, the maximum acoustic operation range is strictly configured to 10.32 m, providing a high range resolution of 7.5 mm. The sonar operates in profiling mode with a step angle of 1.6° per scan, mechanically rotating to obtain echo distances of the surrounding environment. Operating over a high-speed serial interface, the motor step time is optimized to 35 ms per beam, resulting in a full 360-degree scan period of approximately 7.9 s. This significant time delay spanning nearly 8 s is the primary physical cause of the severe temporal point cloud deformation addressed in this study. The second is a dual-compass attitude measurement system: one compass is placed horizontally and the other vertically downward, each capable of outputting yaw, pitch, and roll angles. This industrial-grade system outputs synchronized attitude data at an update frequency of 20 Hz, offering an angular resolution of 0.01 degrees and a high dynamic accuracy of approximately ±0.2 degrees for pitch and roll, and ±0.8 degrees for heading. This continuous attitude output provides the measurement basis for beam-level attitude association during the 7.9 s slow-scanning period. Since the sonar scanning plane is horizontal and the horizontally placed compass has its coordinate axes parallel to the sonar installation orientation, the yaw, pitch, and roll angles from the horizontal compass are uniformly used for compensation calculations to ensure the accuracy of spatial attitude projection.

To evaluate the proposed algorithm under controlled severe-motion conditions, a real-data-driven semi-physical disturbance-injection strategy based on three-dimensional geometric transformations is adopted. Unlike pure digital simulation without measured background characteristics, this strategy uses sonar echo data acquired under steady-state pool conditions as the baseline, thereby retaining the hardware noise and multipath effects present in the measured data. At the software level, a sinusoidal roll disturbance with an amplitude of ±45° and a frequency of 0.1 Hz is injected into the measured compass time series, and a constant planar drift velocity is superimposed. The specific drift velocities used in the single-scan and continuous-sequence experiments are described separately in Section 4.4 and Section 4.5. Following the geometric projection relationship of acoustic ranging, the range stretching and spatial misalignment caused by the injected motion are reconstructed. This strategy provides controlled disturbance data for quantitative evaluation while retaining the background characteristics of the measured pool data. The resulting tests evaluate the algorithm under prescribed periodic disturbances and do not constitute complete physical validation under real extreme sea conditions. Aperiodic and high-frequency vibrations caused by propulsion systems and turbulent currents are not fully represented by the current disturbance model and may affect the compensation performance in actual deployments.

To ensure the reproducibility of the proposed method, the key algorithm parameters used in all experiments are summarized in Table 1. Rather than using exhaustive grid search, these parameters were determined through a combination of physical constraint analysis and empirical tuning based on the baseline steady-state dataset.

Specifically, for the probabilistic weighting model, the Gaussian decay width (σ = 7.07°) was calibrated according to the maximum natural pitch and roll variance observed during the robot’s steady-state hovering. This ensures that normal operational sway is tolerated, while extreme tilts that have a high probability of generating multipath reflections are strictly penalized. The minimum confidence value ($w_{min}$) was empirically chosen to aggressively suppress noise by 90% while preventing singularity in the Weighted ICP matrix solver.

For the lightweight SLAM back-end, the keyframe threshold (ΔS = 0.2 m) and the sub-map search radius (R = 4.0 m) were configured based on the 6 m × 3 m confined pool dimensions and the robot’s low-speed operational profile. This configuration guarantees sufficient feature overlap while culling redundant hovering data. Finally, the EMA smoothing coefficient (β = 0.6) was tuned to retain 60% of the historical velocity inertia, achieving an optimal balance between responsiveness to physical acceleration and robustness against high-frequency matching noise.

### 4.2. Evaluation Metrics

To quantitatively evaluate the compensation and registration results, three evaluation metrics are introduced: Maximum Absolute Error MaxAE, Mean Absolute Error MAE, and Registration Root Mean Square Error Registration RMSE. For the single-scan deformation experiment, the steady-state point cloud acquired under calm-water conditions is used as the reference. Let $p_{i}$ denote the i-th evaluated point and let p^i denote its nearest-neighbor point in the steady-state reference point cloud, with the total number of evaluated points being *N*. The maximum absolute error is defined as the maximum of the absolute errors over all sampling points, as shown in Equation (10):

(10) MaxAE=maxi=1N∥pi−p^i∥2

This metric reflects the limit of perception deviation under harsh operating conditions and evaluates the most severe outward bulging or inward indentation deformation of the environmental point cloud contour. It directly relates to the safety margin for obstacle avoidance and wall-contact operations of the robot in confined waters. The mean absolute error is defined as the average of the absolute errors over all sampling points, as shown in Equation (11):

(11) MAE=1N∑i=1N∥pi−p^i∥2

This metric measures the overall point-cloud deformation over the evaluated scan and reflects the geometric consistency of the compensated point cloud relative to the steady-state reference point cloud. Both of the above metrics are calculated from the point-to-reference distances between the evaluated point cloud and the steady-state reference point cloud; smaller values indicate better deformation compensation performance.

Furthermore, the Registration Root Mean Square Error, Registration RMSE, is used to quantify the internal scan-to-map registration residual of the different SLAM frameworks. After applying the estimated planar translation, the registration residual between the current scan and the corresponding target map is calculated as follows:

(12) $$Registration\;RMSE=\sqrt{\frac{\sum_{j=1}^{M}w_{j}\|p_{j}^{src}+t^{*}-p_{j}^{tgt}{\|}_{2}^{2}}{\sum_{j=1}^{M}w_{j}}}$$

Here, M is the number of inlier correspondences retained during registration, $p_{j}^{src}$ and $p_{j}^{tgt}$ are the two-dimensional planar coordinates of the corresponding source and target points, respectively, and $t^{*}$ is the planar translation estimated by ICP registration. For the traditional navigation odometry SLAM and the 3D attitude compensation SLAM without temporal correction, all retained correspondences are assigned equal weights, namely $w_{j}$ = 1, and Equation (12) reduces to the standard unweighted RMSE. For the proposed confidence-weighted ICP, $w_{j}$ denotes the attitude-derived confidence weight of the j-th source point. 

### 4.3. Evaluation of Front-End Processing Using Real Pool Data Under Basic Operating Conditions

**Figure 4:**
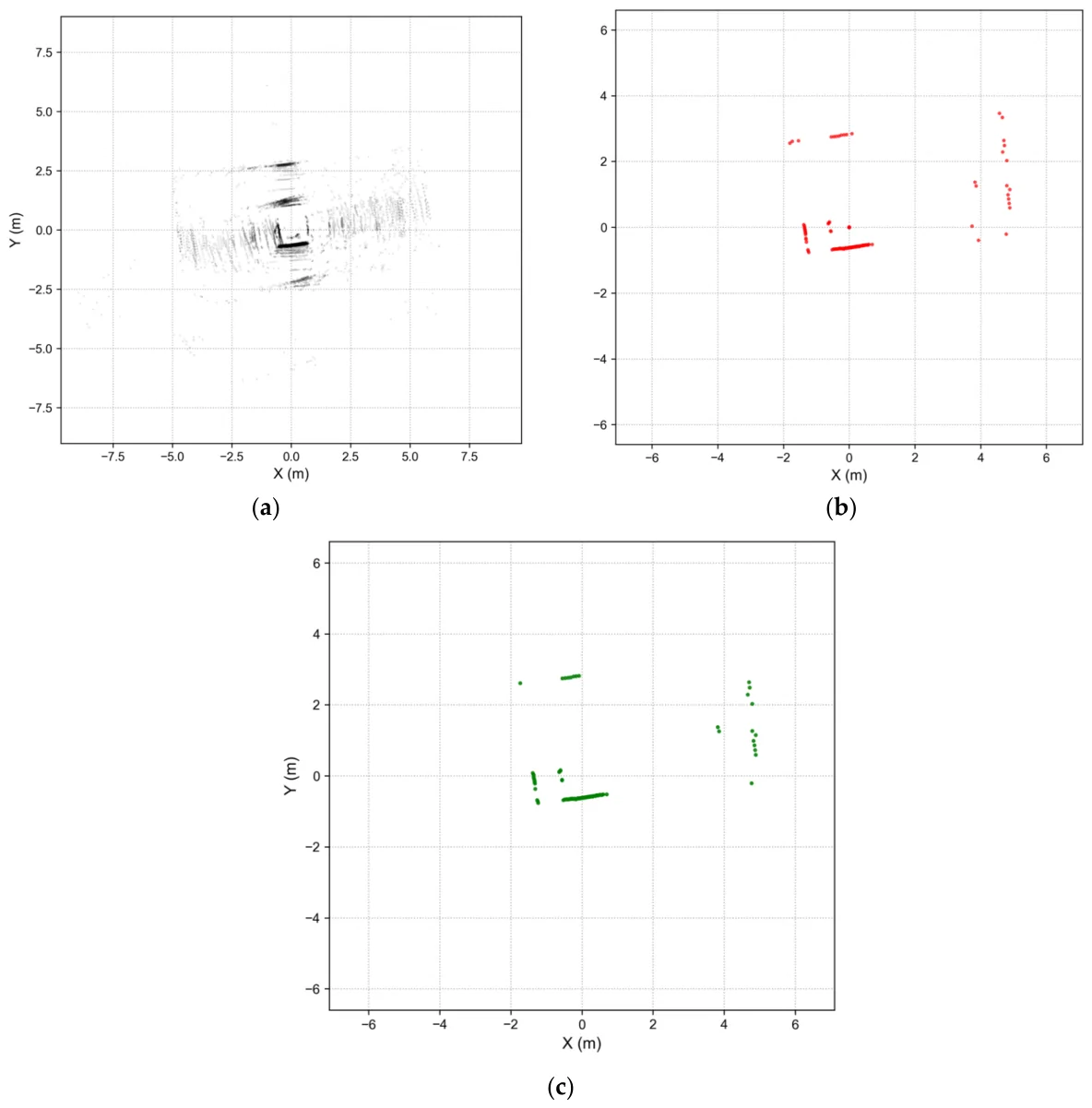
Comparison of point cloud distributions under different conditions. (**a**) Full raw point cloud projection, (**b**) Raw point cloud of a single scan, (**c**) Effective point cloud after filtering by the proposed algorithm.

To evaluate the denoising and feature-preservation capabilities of the front-end acoustic filtering and geometry-based validity screening under real confined-water sensing conditions, a feature extraction comparison experiment was conducted using the original pool dataset acquired under steady-state conditions. Figure 4 shows the raw acoustic data distribution and the corresponding front-end feature extraction results. Figure 4a presents the projection of the complete raw point cloud collected during the test period. These points are geometric projections of the range values directly output by the sonar hardware. The unprocessed point cloud contains numerous scattered echoes from suspended particles and false boundary structures caused by acoustic multipath interference. These structural artifacts can adversely affect subsequent SLAM registration.

To present the front-end filtering details more clearly, Figure 4b and Figure 4c show the unfiltered point cloud from one complete scan cycle and the effective feature point cloud retained after front-end processing, respectively. After valid-range truncation, local median-based spike filtering, and geometry-based adaptive masking, scattered outliers and part of the multipath artifacts are suppressed, and the pool-wall boundary becomes more concentrated. For the selected complete scan cycle, the number of retained beams decreases from 225 to 98, corresponding to a feature retention rate of 43.56%. Since multipath false targets can introduce erroneous correspondences during registration, the front-end processing prioritizes the suppression of unreliable measurements while retaining sufficient geometric features. Figure 4c shows that, under the evaluated basic operating condition, the proposed front-end processing improves the clarity and concentration of the pool-wall contour. These results demonstrate the effectiveness of the acoustic filtering and geometry-based validity screening for preprocessing real pool data.

### 4.4. Ablation and Continuous-Time Baseline Comparison Under Injected Disturbance Conditions

To verify the underlying correction capability of the beam-level three-dimensional spatiotemporal joint compensation algorithm under severe swaying and translational drift conditions, an ablation experiment over a complete sonar scan cycle was conducted on semi-physical data injection simulation data with ±45° attitude disturbance and translational drift. To ensure objectivity, a 10% velocity estimation lag error from the SLAM back-end was simulated when testing the proposed algorithm. For clarity, the specific implementations of the baseline algorithms used in this comparison are explicitly defined. The traditional two-dimensional static projection baseline is implemented by placing all acoustic beams at the starting pose of the current scan frame, completely ignoring any intra-scan translation or attitude tilt. The beam-level constant-velocity translational compensation baseline is implemented by interpolating the planar displacement for each beam using the velocity feedback, but it ignores the spatial tilt caused by pitch and roll. In addition, a representative continuous-time baseline adapted from Furgale et al. [11] was implemented using cubic temporal basis functions. Seven uniformly distributed pose observations within the scan period were used to fit the continuous planar displacement trajectory, with the same 10% displacement-scale lag and an additional zero-mean Gaussian observation noise of 0.01 m. The fitted trajectory was then used for beam-level translational compensation after applying the same three-dimensional attitude flattening. Table 2 presents the comparison of point-to-reference errors for five compensation strategies under the same injected disturbance conditions.

The adapted continuous-time cubic B-spline method based on Furgale et al. [11] achieves a MaxAE of 0.102 m and an MAE of 0.045 m. Under the same injected disturbance conditions, the proposed spatiotemporal joint compensation method yields slightly lower values of 0.098 m and 0.044 m, respectively. The two methods therefore provide comparable deformation-correction accuracy, while the proposed method achieves marginally smaller point-to-reference errors using a lightweight velocity-feedback formulation without batch estimation of continuous-time spline coefficients. In terms of both maximum point-to-reference error MaxAE and mean point-to-reference error MAE, the proposed spatiotemporal joint compensation strategy achieves the best deformation compensation results, reducing MaxAE to 0.098 m and MAE to 0.044 m. In contrast, the traditional 2D static projection without any compensation suffers severe degradation, with the point cloud exhibiting significant outward bulging and helical misalignment, resulting in a MaxAE of 1.059 m. Further analysis shows that the single-dimensional strategies (beam-level constant-velocity translational compensation alone) still yield extreme errors between 0.85 m and 0.99 m compared with the uncompensated baseline. These results indicate that for single-beam mechanical scanning sonar, spatial attitude deformation and temporal slow-scanning deformation are coupled; compensation in only one dimension cannot meet the safe obstacle avoidance requirements in confined waters. By combining three-dimensional attitude flattening and beam-level temporal back-estimation, the proposed method demonstrates the effectiveness of spatiotemporal joint compensation in eliminating nonlinear motion deformation.

### 4.5. Comparative Evaluation of Continuous Trajectory Estimation and Registration Consistency Under Complex Disturbances

To evaluate the registration stability and internal trajectory consistency of the proposed algorithm over an extended disturbance sequence, a disturbance dataset containing 43 full scan cycles with superimposed extreme roll and strong current drift was used to compare the continuous trajectory estimates and mean registration RMSE values of three SLAM frameworks. Specifically, the traditional navigation odometry SLAM baseline builds the local map directly using the uncompensated two-dimensional static projection point clouds. The 3D attitude compensation SLAM baseline without temporal correction performs beam-level slant-range flattening using the pitch and roll measurements associated with each beam, but does not compensate for translational displacement accumulated during the scan. These comparative baselines are not based on specific proprietary frameworks but are explicitly implemented as standard dead-reckoning compensations utilizing velocity feedback from the back-end, representing typical methods widely adopted in standard autonomous underwater vehicle SLAM systems. Table 3 presents the comparison of mean registration RMSE values of different SLAM algorithms under complex disturbances.

From the perspective of the registration root-mean-square error RMSE, the proposed dynamic keyframe SLAM achieves the best registration consistency, with a mean registration RMSE of 0.158 m. Further analysis shows that the traditional navigation odometry SLAM, which does not compensate for dynamic deformation, yields a mean registration RMSE of 0.275 m and exhibits marked registration drift. In contrast, the 3D attitude compensation SLAM without temporal correction corrects attitude-induced spatial range stretching but does not compensate for the translational displacement accumulated during the slow-scanning process. Consequently, its registration consistency is only slightly improved, with a mean registration RMSE of 0.264 m.

In comparison, the proposed method integrates beam-level spatiotemporal joint compensation, dynamic local sub-map extraction, and confidence-weighted ICP registration. As further examined in Section 4.6, the measurable reduction in the mean internal registration residual is mainly associated with spatiotemporal compensation and the dynamic closed-loop mapping mechanism, while confidence weighting serves as an auxiliary robustness safeguard for low-confidence correspondences. Even under the continuous ±45° roll disturbance, the proposed method yields a mean internal registration residual of 0.158 m. This reported residual value is approximately 42.5% lower than the value of 0.275 m obtained by the traditional navigation odometry SLAM under its corresponding registration formulation. The registration residual remains bounded throughout the tested sequence, indicating improved scan-to-submap consistency and better internal consistency of the estimated trajectory under the evaluated conditions.

To validate the lightweight and real-time claims, the computational efficiency of the algorithm was evaluated. The proposed framework is entirely CPU-based and does not require GPU acceleration. On a standard embedded Intel Core i5 processor, the average processing time for a complete scan containing 225 beams is strictly less than 50 milliseconds. Given that the physical acoustic scanning period takes 7.9 s, the algorithmic processing consumes less than one percent of the available time window. This low computational overhead indicates that the method has the potential for real-time deployment on low-power underwater robot platforms.

### 4.6. Module-Level Ablation Analysis of the Proposed Framework

To further isolate the contributions of the principal components in the complete framework, a module-level ablation experiment was conducted using the same disturbance sequence, valid scan cycles, initialization conditions, and registration parameters as those used in Section 4.5. Four configurations were evaluated: the complete method, the method without confidence weighting, the method without beam-level spatiotemporal compensation, and the method without the dynamic local sub-map and closed-loop velocity feedback.

For the configuration without confidence weighting, all retained point correspondences were assigned an equal weight of one during ICP registration, while the point-cloud construction, beam-level motion compensation, dynamic local sub-map, and velocity-feedback procedures remained unchanged. For the configuration without beam-level spatiotemporal compensation, the uncompensated two-dimensional projection point cloud was passed to the same dynamic keyframe SLAM back-end. For the configuration without the dynamic local sub-map and velocity feedback, an accumulated global map was used instead of the local keyframe sub-map, and the feedback velocity was set to zero. The mean internal registration residual was used as the evaluation metric. The results are presented in Table 4.

The complete method yields a mean internal registration residual of 0.158 m. Removing beam-level spatiotemporal compensation increases the residual to 0.185 m. This result indicates that the beam-wise correction of attitude-induced slant-range deformation and intra-scan translational displacement contributes directly to improved scan-to-submap consistency. When the dynamic local sub-map and closed-loop velocity feedback are removed, the residual further increases to 0.221 m, demonstrating that local-map selection and feedback-based motion prediction play important roles in maintaining continuous registration stability.

Removing confidence weighting results in a mean internal registration residual of 0.158 m, which is nearly identical to that of the complete method. This limited difference is mainly associated with the characteristics of the present controlled disturbance sequence. Under the rigid pool-wall environment and sinusoidal attitude disturbance, most retained sonar measurements have high geometric confidence, with a mean frame confidence weight greater than 0.998. Consequently, the soft weighting matrix is close to a uniform weighting matrix, and the weighted ICP approaches standard ICP in this structured case.

This result does not indicate that the confidence-weighting mechanism is mathematically redundant. Instead, it shows that its effect is condition-dependent. The mechanism is designed as a lightweight robustness safeguard rather than as the dominant source of mean-error reduction under all operating conditions. When low-confidence measurements are rare, it introduces almost no measurable change to the registration result. When unreliable correspondences occur, the weighting mechanism provides a means of reducing their contribution without changing the ICP structure or introducing an additional outlier-rejection stage. Its effectiveness under environments containing a higher proportion of multipath echoes, flexible boundaries, suspended particles, or bubbles remains to be further evaluated using physical measurements.

### 4.7. Comparative Analysis of Motion Compensation Visualization Results

**Figure 5:**
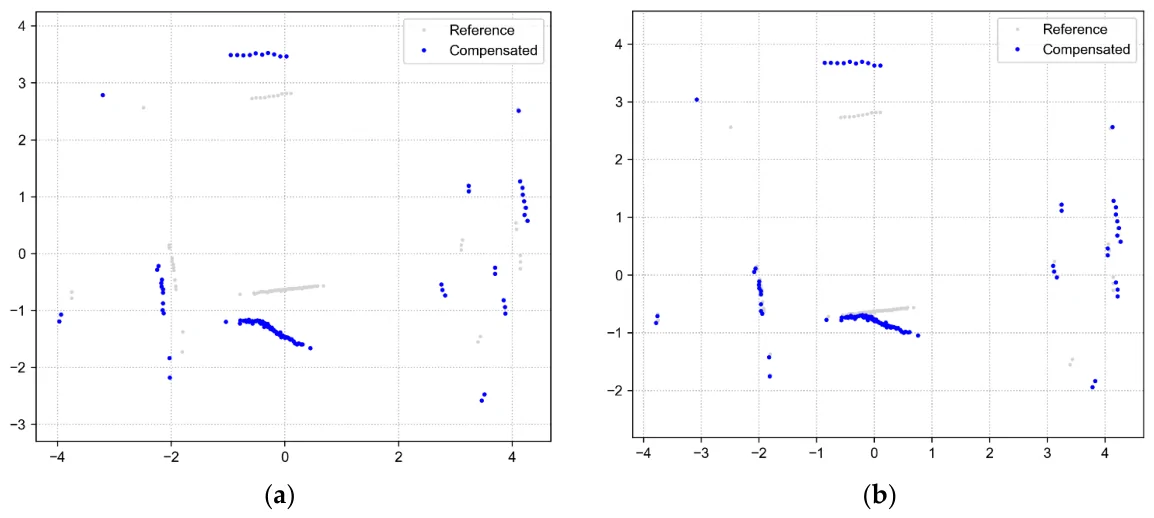

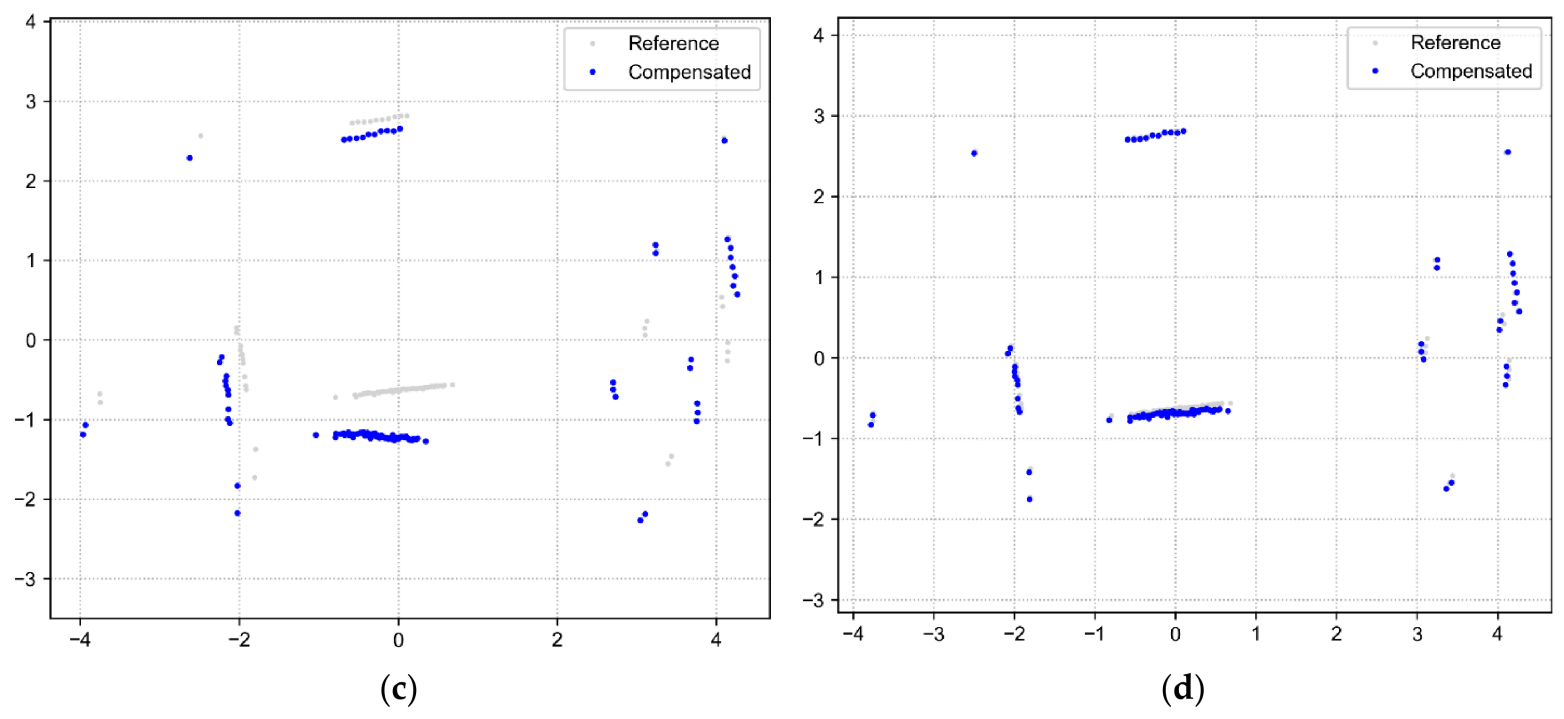
Motion compensation visualization results. (**a**) Traditional 2D static projection (no compensation), (**b**) Beam-level constant-velocity translational compensation only, (**c**) 3D spatial attitude compensation only, (**d**) Proposed spatiotemporal joint compensation. The gray points denote the steady-state reference point cloud, while the blue points denote the point cloud processed using the corresponding compensation method.

The proposed beam-level spatiotemporal joint deformation compensation algorithm can more effectively eliminate the severe deformation of acoustic point clouds under the injected disturbance conditions. Figure 5 shows the point cloud correction visualization results of four different compensation strategies within a single complete sonar scan cycle in the ablation experiment.

In each subplot, the gray points represent the steady-state reference point cloud acquired directly by the sonar during a baseline scan under calm-water conditions, while the blue points represent the point cloud processed using the corresponding compensation method. Using the steady-state reference point cloud rather than idealized straight lines preserves the natural sparsity of the acoustic echoes, the actual reflection thickness of the pool walls, and the inherent artifacts present in the measured data.

It can be observed that the proposed algorithm in subplot d achieves the smallest visible deformation. The blue point cloud closely follows the gray reference point cloud and recovers a pool-wall contour consistent with the reference measurement. In contrast, subplot a, which does not apply motion compensation, exhibits marked outward bulging due to the combined effects of platform swaying and slow scanning. Subplot b reduces temporal misalignment but retains arc-shaped stretching because three-dimensional attitude correction is not applied. Subplot c corrects attitude-induced range stretching but does not compensate for the displacement accumulated during scanning, resulting in an overall offset relative to the reference point cloud.

These results show that the proposed spatiotemporal joint deformation compensation method reduces nonlinear range stretching through three-dimensional attitude-based slant-range correction and reduces slow-scanning misalignment through beam-level displacement compensation based on velocity feedback. The resulting point cloud therefore exhibits better geometric consistency with the steady-state reference measurement.

Unlike the signal-level phase compensation developed for synthetic aperture sonar arrays [12], which maintains coherence across multi-hydrophone arrays through sub-beam processing, our method addresses a fundamentally different problem arising from single-beam mechanical scanning sonar. The slow-scanning nature of this sonar (7.9 s per revolution) introduces macroscopic geometric deformations, including helical misalignment and nonlinear range stretching, rather than phase incoherence. Our beam-level approach directly corrects these geometric distortions at the point-cloud level, bypassing the computationally expensive signal-level phase operations required in [12]. Moreover, by leveraging the velocity-prior feedback loop from the lightweight SLAM backend, the proposed method achieves efficient temporal displacement compensation with an average per-beam processing time well below the 35 ms motor step interval, enabling real-time deployment on low-power underwater robots without specialized hardware acceleration.

## 5. Conclusions

When underwater robots use single-beam sonar to operate in confined waters such as ponds and aquaculture cages, they are susceptible to multipath interference in complex underwater environments. Moreover, the combination of robot swaying and the slow-scanning characteristic of mechanical sonar causes marked range stretching and point-cloud deformation, which reduces the reliability of conventional scan registration methods. This paper proposes a motion deformation compensation and localization method for single-beam sonar. First, an attitude-derived probabilistic confidence model is introduced to characterize the geometric reliability of retained sonar measurements and reduce the contribution of low-confidence correspondences during weighted registration. Second, a beam-level spatiotemporal joint deformation compensation algorithm is designed, which reduces swaying deformation and slow-scanning misalignment through spatial slant-range flattening and beam-level displacement back-estimation based on beam time offsets and feedback velocity. Finally, a lightweight SLAM architecture integrating keyframe-based dynamic sub-maps is constructed. Confidence-weighted ICP incorporates the geometric confidence scores as an auxiliary robustness term, while the estimated planar velocity is fed back to the temporal compensation module to form a closed-loop correction process.

Experimental results obtained from the real-data-driven semi-physical disturbance tests show that, under the injected ±45° roll disturbance and translational drift, the proposed method reduces the maximum point-to-reference error MaxAE from 1.059 m to 0.098 m. The reported mean internal registration residual decreases from 0.275 m for the traditional navigation odometry SLAM to 0.158 m for the proposed method, corresponding to a numerical reduction of approximately 42.5%. The proposed method therefore reduces point-cloud deformation and improves continuous registration stability under the evaluated disturbance conditions.

Localization in confined waters is important for reducing collision risks and improving the efficiency of underwater cleaning and harvesting operations. In the present study, real pool measurements are used to evaluate the front-end processing under authentic hardware noise and multipath interference, while the severe-motion compensation and continuous registration tests are based on controlled disturbance injection using the measured pool data. Therefore, the current results do not constitute complete physical validation under real extreme sea conditions. Actual aquaculture environments may contain aperiodic and high-frequency vibrations caused by propulsion systems and turbulent currents, which are not fully represented by the present disturbance model. Future work will conduct physical motion experiments in dynamic pools and operational aquaculture environments, collect multi-source measured datasets, and investigate multi-sensor fusion using sonar, vision cameras, and Doppler velocity logs.

## Figures and Tables

**Table 1:** Key parameters of the proposed spatiotemporal motion compensation and SLAM algorithm.

Parameter Category	Parameter Name	Symbol	Value
Confidence Weighting	Gaussian decay width	σ	7.07°
Confidence Weighting	Minimum confidence value	$w_{min}$	0.1
Dynamic SLAM	Keyframe spatial displacement threshold	ΔS	0.2 m
Dynamic SLAM	Local sub-map search radius	R	4.0 m
Dynamic SLAM			
Velocity Feedback	EMA smoothing coefficient	β	0.6
Acoustic Filtering	Valid echo distance range	D	0.5 m–6.5 m

**Table 2:** Comparison of point-to-reference errors for the ablation strategies and continuous-time baseline.

Compensation Strategy	MaxAE/(m)	MAE/(m)
Traditional 2D static projection (no compensation)	1.059	0.564
3D spatial attitude compensation only	0.858	0.428
Beam-level constant-velocity translational compensation only	0.988	0.219
Continuous-time cubic B-spline	0.102	0.045
Proposed spatiotemporal joint compensation	0.098	0.044

**Table 3:** Comparison of mean internal registration residuals reported by different SLAM frameworks.

Localization Algorithm Framework	Mean Internal Registration Residual/m
Traditional navigation odometry SLAM	0.275
3D attitude compensation SLAM without temporal correction	0.264
Proposed dynamic keyframe SLAM	0.158

**Table 4:** Module-level ablation results of the proposed framework under the same disturbance sequence.

Configuration	Mean Internal Registration Residual/m
Full method	0.158
Without confidence weighting	0.158
Without beam-level spatiotemporal compensation	0.185
Without dynamic local sub-map and velocity feedback	0.221

## Data Availability

Enquiries regarding the experimental data should be made by contacting the first author.
